# Validation of the transplant conditioning intensity (TCI) index for allogeneic hematopoietic cell transplantation

**DOI:** 10.1038/s41409-023-02139-5

**Published:** 2023-11-17

**Authors:** Alexandros Spyridonidis, Myriam Labopin, Tobias Gedde-Dahl, Arnold Ganser, Matthias Stelljes, Charles Craddock, Eva Maria Wagner-Drouet, Jurjen Versluis, Thomas Schroeder, Igor Wolfgang Blau, Gerald. G. Wulf, Peter Dreger, Gitte Olesen, Henrik Sengeloev, Nicolaus Kröger, Victoria Potter, Edouard Forcade, Jakob Passweg, Régis Peffault de Latour, Johan Maertens, Keith M. O. Wilson, Jean Henri Bourhis, Juergen Finke, Eolia Brissot, Ali Bazarbachi, Sebastian Giebel, Bipin P. Savani, Arnon Nagler, Fabio Ciceri, Mohamad Mohty

**Affiliations:** 1 Bone Marrow Transplantation Unit and Institute of Cellular Therapy, University of Patras, Patras, Greece; 2 EBMT Unit, Sorbonne University, Saint-Antoine Hospital, AP-HP, INSERM UMRs 938, Paris, France; 3 Oslo University Hospital, Rikshospitalet Clinic for Cancer Medicine, Hematology Dept. Section for Stem Cell Transplantation, Oslo, Norway; 4 Hannover Medical School, Department of Haematology, Hemostasis, Oncology and Stem Cell Transplantation, Hannover, Germany; 5 University of Muenster, Dept. of Hematol./Oncol., Muenster, Germany; 6 University Hospital Birmingham NHSTrust, Queen Elizabeth Medical Centre, Edgbaston, Dept. of Haematology, Birmingham, United Kingdom; 7 University Medical Center Mainz, Department of Hematology, Oncology and Pneumology, Mainz, Germany; 8 Erasmus MC Cancer Institute, University Medical Center Rotterdam, Department of Hematology, Rotterdam, Netherlands; 9 University Hospital Essen, Dept. of Bone Marrow Transplantation, Essen, Germany; 10 Charité Universitätsmedizin Berlin, Onkologie und Tumorimmunologie, Berlin, Germany; 11 Universitaetsklinikum Goettingen, Abteilung Hämatologie und Onkologie, Goettingen, Germany; 12 University of Heidelberg, Medizinische Klinik u. Poliklinik V, Heidelberg, Germany; 13 Aarhus University Hospital, Aarhus, Denmark; 14 National University Hospital, Bone Marrow Transplant Unit, Copenhagen, Denmark; 15 University Hospital Eppendorf, Bone Marrow Transplantation Centre, Hamburg, Germany; 16 Kings College Hospital, Dept. of Haematological Medicine, King’s Denmark Hill Campus, London, United Kingdom; 17 Hôpital Haut-leveque, CHU Bordeaux, Pessac, France; 18 University Hospital Basel, Dept of Hematology, Basel, Switzerland; 19 Hopital St. Louis, Dept. of Hematology-BMT, Paris, France; 20 University Hospital Gasthuisberg Dept. of Hematology, Leuven, Belgium; 21 University Hospital of Wales, Department of Haematology, Cardiff, United Kingdom; 22 Gustave Roussy Cancer Campus, Department of Hematology, Villejuif, France; 23 University of Freiburg, Department of Hematology/Oncology, Freiburg, Germany; 24 Hospital Saint Antoine, Department of Hematology, Paris, France; 25 American University of Beirut-Medical Center, Department of Internal Medicine-Bone Marrow Transplantation Program, Beirut, Lebanon; 26 Maria Sklodowska-Curie National Research Institute of Oncology, Department of Hematology, Gliwice, Poland; 27 Vanderbilt University Medical Center, Department of Hematology, Nashville, TN USA; 28 Chaim Sheba Medical Center, Department of Hematology, Tel-Hashomer, Israel; 29 Hematology Division, Ospedale San Raffaele s.r.l., Haematology and BMT, Milano, Italy

**Keywords:** Therapeutics, Stem-cell research

## Abstract

The intensity of the conditioning regimen given before allogeneic hematopoietic cell transplantation (allo-HCT) can vary substantially. To confirm the ability of the recently developed transplant conditioning intensity (TCI) score to stratify the preparative regimens of allo-HCT, we used an independent and contemporary patient cohort of 4060 transplant recipients with acute myeloid leukemia meeting inclusion criteria from the discovery study (allo-HCT in first complete remission, matched donor), but who were allografted in a more recent period (2018–2021) and were one decade older (55–75 years, median 63.4 years), we assigned them to a TCI category (low *n* = 1934, 48%; intermediate *n* = 1948, 48%, high *n* = 178, 4%) according to the calculated TCI score ([1–2], [2.5–3.5], [4–6], respectively), and examined the validity of the TCI category in predicting early non-relapse mortality (NRM), 2-year NRM and relapse (REL). In the unadjusted comparison, the TCI index provided a significant risk stratification for d100 and d180 NRM, NRM and REL risk. In the multivariate analysis adjusted for significant variables, there was an independent association of TCI with early NRM, NRM and REL. In summary, we confirm in contemporary treated patients that TCI reflects the conditioning regimen related morbidity and anti-leukemic efficacy satisfactorily and across other established prognostic factors.

## Introduction

The intensity of the conditioning regimen given before allogeneic hematopoietic cell transplantation (allo-HCT) can vary substantially, determines acute regimen related toxicity and impacts transplant outcomes. The myeloablative conditioning (MAC) versus the reduced intensity conditioning (RIC) classification has set for the last two decades a global standard to indicate transplant conditioning intensity and proved a reliable approach for clinical decisions and registry analyses [1, 2]. As intensity represents a continuum and novel drugs and new conditioning regimens are now used, with some of them not being readily amenable to the RIC/MAC nomenclature [3–7], we recently developed a tool which provided finer stratification, better discriminating ability and more standardized assessment of the intensity of the preparative regimen [8]. Briefly, we assigned intensity weight scores for frequently used components in the conditioning regimen, we used their sum to generate the transplant conditioning intensity (TCI) score, and we built a discrete 3-category stratification TCI index which was tested on a discovery cohort of 8255 patients with acute myeloid leukemia (AML) allografted between 2005 and 2017. TCI group assignment (low, intermediate, high) was the most important determinant of day (d) 100 and d180 early non-relapse mortality (NRM) and was very effective in predicting 2-year NRM and relapse (REL), independently from other established prognostic factors. The internal validity of the TCI model was assessed using a bootstrapping technique, however, a formal validation conducted in a separate and more contemporary patient population was lacking, hence the current validation study. Using data reported to the European Society for Blood and Marrow Transplantation (EBMT) registry, we included transplant recipients meeting inclusion criteria from the discovery study but who were allografted in a more recent period (2018 to June 2021), we assigned them to a TCI category (low, intermediate, high) according to the calculated TCI score ([1,2], [2.5–3.5], [4–6], respectively), as previously described [8], and examined the validity of the TCI category in predicting early NRM, 2-year NRM and REL.

## Materials and methods

### Study design and data collection

This is a retrospective, multicenter, registry-based analysis. Data were provided by the EBMT registry, to which >600 transplant centers submit annually anonymized data of all their consecutive HCTs according to specific guidelines and audited quality measures, following patient informed consent and according to the local regulations applicable at the time of transplantation. The Acute Leukemia Working Party (ALWP) of the EBMT approved the study in accordance with the guidelines of the Declaration of Helsinki. We included patients with AML between 55 and 75 years of age who had received an allogeneic HCT at first complete remission between January 2018 and June 2021. Other inclusion criteria included availability of detailed conditioning information, time from diagnosis to HCT < 18 months, use of peripheral blood stem cell (PBSC) or bone marrow (BM) grafts from a matched sibling or HLA-matched unrelated donor. Cases with a missing HCT-comorbidity index (HCT-CI) score were excluded (*n* = 464). The TCI score was calculated for every patient by adding the intensity weights for each component given any day before the graft infusion, as shown in Supplementary Table 1, and as previously described [8]. Assignment to the low, intermediate, or high TCI category was performed according to the TCI score of [1,2], [2.5–3.5] and [4–6], as previously described. For example, a regimen consisted of busulphan 12.8 mg/kg iv (3 points) and fludarabine 120 mg/m^2^ (0.5 points) has a TCI score of 3.5 and is assigned as an intermediate TCI regimen, whereas when the same dose busulphan is combined with cyclophosphamide 120 mg/kg as in the classical BuCy protocol the TCI score is 4 (high TCI regimen). Data sharing is available through the ALWP office (myriam.labopin@upmc.fr).

### Endpoints and statistical analysis

The primary endpoint for estimating the impact of TCI was early NRM measured at d100 and d180 from the time of stem cell infusion. Secondary endpoints included NRM and REL incidence at 2 years. NRM was defined as death without evidence of REL. Relapse incidence and NRM were calculated using cumulative incidence curves in a competing risk setting. Overall survival (OS) defined as time to death from any cause, and leukemia-free survival (LFS) defined as time being alive without evidence of REL, were also reported and were calculated from time of transplant using the Kaplan–Meier estimate. Univariate analyses for NRM and REL were performed using Gray’s test. Univariate comparisons between TCI groups were performed using the Chi-squared or Fischer’s exact test for categorical variables and the Kruskal-Wallis test for continuous variables. Multivariate analysis was performed using a Cox proportional-hazards model which included variables differing significantly between the groups, factors known to be associated with outcomes, plus a center frailty effect to take account of the heterogeneity across centers, as previously reported [9]. The results were expressed as the hazard ratios (HR) with 95% confidence interval (CI). All tests were two-sided with the type 1 error rate fixed at 0.05. Statistical analyses were performed with SPSS 27.0 (SPSS Inc., Chicago, IL, USA) and R 4.1.1 (R Development Core Team, Vienna, Austria, URL: https://www.R-project.org/).

## Results

### Characteristics of the validation cohort

The validation cohort comprised 4060 adult patients with AML who were transplanted in first complete remission in the most recent period (median year 2019, range 2018–2021). In contrast to the discovery cohort which included patients between 45 and 65 years of age (median 55.6 years), patients in this validation dataset were one decade older (median 63.4 years, range 55–75). In total, 48 different conditioning regimens were used (Supplementary Table 2). Baseline characteristics are shown by TCI group in Table 1. In this validation cohort, 1934 (48%) and 1948 (48%) patients were assigned to the low, and intermediate TCI group, respectively, while a high TCI was less prevalent (*n* = 178, 4% of patients). As expected, there was an inverse relationship between age and TCI, with a median age of 65 years (interquartile range [IQR], 61.3–68.4), 62 years (IQR, 58.8–65.9) and 59 years (IQR, 56.8–63.3) for the low, intermediate, and high TCI groups, respectively (*p* < 0.0001). About 18–33% of patients among TCI groups had a low (≤80%) Karnofsky Performance Score (KPS) and/or high (≥3) HCT-CI, with patients in the low TCI category more likely to have a lower KPS ≤ 80% and a higher HCT-CI ≥ 3 (*p* < 0.0001). Except for the more frequent use of matched sibling donors in the high TCI cohort (*p* < 0.0001), other characteristics were distributed equally between the 3 TCI groups. The most often used immunosuppressive drug combination for graft versus host disease (GvHD) prophylaxis was cyclosporine/mycophenolate mofetil (34.8%, 31.3% and 31.6%) or cyclosporine/methotrexate (34.9%, 32.5% and 39%), whereas post-transplant cyclophosphamide (PTCY) was used in 8.8%, 11.3% and 13.9% of TCI low, intermediate, and high groups, respectively (Table 1). The median follow-up of survivors was 22.3 months (IQR, 20.8–23.2). The outcomes for the entire population were as follows: cumulative incidence of d100 NRM was 6.2% (95% CI 5.5–7), of d180 NRM was 10.2% (95% CI 9.3–11.2), of 2-year NRM was 19.2% (95% CI 17.8–20.5), of REL was 25.7% (95% CI 24.2–27.3), of acute graft-versus-host disease (GVHD) grades II-IV was 22.1% (95% CI 20.8–23.4), of acute GVHD grades was III-IV 7.6% (95% CI 6.8–8.5), of chronic GVHD was 31.7% (95% CI 30.1–33.4) and of extensive chronic GVHD was 14.2% (95% CI 13–15.5). The estimate of LFS and OS at 2 years was 55.1% (95% CI 53.3–56.9) and 62.2% (95% CI 60.4–63.9), respectively. Graft failure was low and did not differ between TCI groups (*p* = 0.34), results not shown. Causes of death are given in Supplementary Table 3 with original disease the main cause in each TCI category.

**Table 1:** Population baseline characteristics of validation cohort.

		Entire cohort	TCI low	TCI intermediate	TCI high	*P*
*N* (%)		4060 (100%)	1934 (48%)	1948 (48%)	178 (4%)	
Follow-up (mo)	Median (IQR)	22.3 [20.8–23.2]	20.5 [18.2–22.1]	23.2 [21.9–23.7]	25.5 [22.6–29.6]	0.12
Year of transplant	Median (min-max)	2019 (2018–2021)	2019 (2018–2021)	2019 (2018–2021)	2019 (2018–2021)	<0.0001
Type of donor	MSD	1014 (25%)	434 (22.4%)	509 (26.1%)	71 (39.9%)	<0.0001
	UD	3046 (75%)	1500 (77.6%)	1439 (73.9%)	107 (60.1%)	
Age (years)	Median(min-max) [IQR]	63.4 (55–75) [59.6–67.2]	65 (55–75) [61.3–68.4]	62.2 (55–75) [58.8–65.9]	59 (55–71.4) [56.8–63.3]	<0.0001
Cytogenetics	Favorable	117 (3.2%)	41 (2.4%)	65 (3.7%)	11 (7.4%)	0.006
	Intermed	2617 (71.5%)	1265 (72.7%)	1253 (70.8%)	99 (66.4%)	
	Adverse	925 (25.3%)	433 (24.9%)	453 (25.6%)	39 (26.2%)	
	NA/failed	401	195	177	29	
AML	de novo	3311 (81.6%)	1546 (79.9%)	1614 (82.9%)	151 (84.8%)	0.033
	Sec AML	749 (18.4%)	388 (20.1%)	334 (17.1%)	27 (15.2%)	
Female to male	No	3425 (84.7%)	1639 (85.1%)	1640 (84.5%)	146 (82%)	0.54
	Yes	621 (15.3%)	288 (14.9%)	301 (15.5%)	32 (18%)	
	Missing	14	7	7	0	
Cell source	BM	146 (3.6%)	53 (2.7%)	85 (4.4%)	8 (4.5%)	0.02
	PB	3914 (96.4%)	1881 (97.3%)	1863 (95.6%)	170 (95.5%)	
In vivo TCD	No	1059 (26.1%)	550 (28.5%)	458 (23.5%)	51 (28.8%)	0.001
	Yes	2991 (73.9%)	1378 (71.5%)	1487 (76.5%)	126 (71.2%)	
	Missing	10	6	3	1	
PTCY	No	3618 (89.8%)	1756 (91.2%)	1719 (88.7%)	143 (86.1%)	0.011
	Yes	410 (10.2%)	169 (8.8%)	218 (11.3%)	23 (13.9%)	
	Missing	32	9	11	12	
KPS	≤80	1162 (30%)	609 (32.9%)	522 (28.3%)	31 (17.8%)	<0.0001
	≥90	2706 (70%)	1240 (67.1%)	1323 (71.7%)	143 (82.2%)	
	Missing	192	85	103	4	
HCT-CI	HCT-CI = 0	1741 (42.9%)	821 (42.5%)	821 (42.1%)	99 (55.6%)	0.0002
	1 or 2	1100 (27.1%)	491 (25.4%)	570 (29.3%)	39 (21.9%)	
	≥3	1219 (30%)	622 (32.2%)	557 (28.6%)	40 (22.5%)	
Patient CMV	Neg	1205 (29.9%)	594 (31%)	571 (29.4%)	40 (22.5%)	0.053
	Pos	2831 (70.1%)	1325 (69%)	1368 (70.6%)	138 (77.5%)	
	Missing	24	15	9	0	
Donor CMV	Neg	1805 (44.9%)	891 (46.5%)	842 (43.6%)	72 (40.9%)	0.11
	Pos	2218 (55.1%)	1025 (53.5%)	1089 (56.4%)	104 (59.1%)	
	Missing	37	18	17	2	
Conditioning	MAC	1221 (30.1%)	123 (6.4%)	930 (47.7%)	168 (94.4%)	<0.0001
	RIC	2839 (69.9%)	1811 (93.6%)	1018 (52.3%)	10 (5.6%)	

Abbreviations for all tables: *AML* acute myeloid leukemia, *CMV* cytomegalovirus, Cytogenetics cytogenetic risk according to MRC classification (Blood 2010; 116:354–65), *BM* bone marrow, *d* day after HCT, *HCT* hematopoietic cell transplantation, *HCT-CI* HCT comorbidity index, *IQR* interquartile range, Intermed intermediate, *KPS* Karnofsky Performance Status, *MAC* myeloablative conditioning, *MSD* matched sibling donor, *mo* months, *NA* not available, *NRM* non-relapse mortality, *PB* peripheral blood, *PTCY* post-transplant cyclophosphamide, *Ref* reference group, *REL* relapse, *RIC* reduced intensity conditioning, *Sec* secondary, *TCD* T-cell depletion, *TCI* transplant conditioning intensity, *UD* unrelated donor.

### Validation of TCI for NRM

**Fig. 1:**
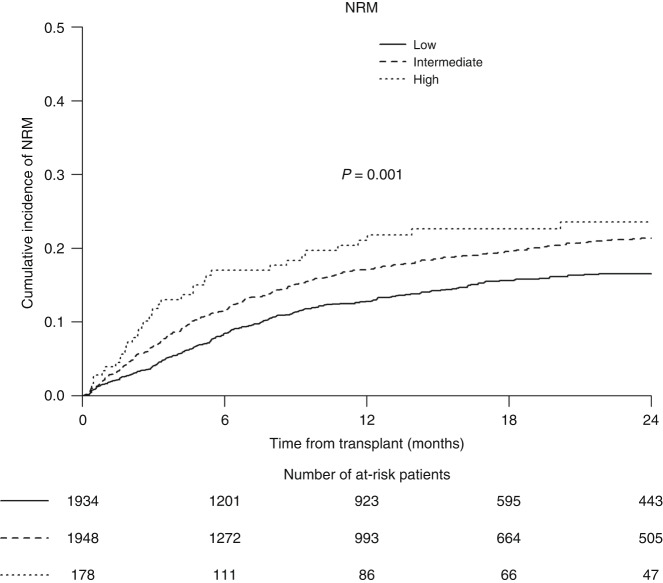
NRM by TCI category. Non-relapse mortality (NRM) for entire validation cohort stratified by Transplant Conditioning Intensity (TCI) category (low, intermediate, high).

The risk of NRM in the validation group followed the same pattern as in the discovery cohort, with a monotonic increase in NRM rate from lower to higher TCI (Fig. 1). In the unadjusted comparison, the TCI provided a highly significant risk stratification for d100, d180 and 2-year NRM, with the cumulative incidences being 4.5% (95% CI, 3.7–5.5), 8.2% (95% CI, 7–9.6) and 16.5% (95% CI, 14.7–18.5) in the low TCI group, rising to 7.3% (95% CI, 6.2–8.5), 11.6% (95% CI, 10.1–13.1) and 21.4% (95% CI, 19.4–23.5) in the intermediate TCI group, and further increasing to 12.4% (95% CI, 8.1–17.8), 17% (95% CI, 11.8–23.1) and 23.5% (95% CI, 17.2–30.5) in the high TCI group, respectively (*p* < 0.0001 for all comparisons) (Table 2). In a multivariable model including baseline characteristics known to impact NRM such as age, KPS, and HCT-CI score (complete case analysis *n* = 3791), TCI group assignment was found to be strongly and independently associated with NRM (Table 3). Relative to the low TCI group, the HRs for d100, d180 and 2-year NRM in the intermediate TCI group were 1.95 (95% CI 1.42–2.69, *p* < 0.0001), 1.62 (95% CI 1.26–2.08, *p* < 0.0001) and 1.44 (95% CI 1.20–1.74, *p* < 0.0001), and in the high TCI group were 4.00 (95% CI 2.2–7.28, *p* < 0.0001), 2.86 (95% CI 1.76–4.64, *p* < 0.0001) and 1.87 (95% CI 1.25–2.80, *p* = 0.003), respectively. In a pairwise comparison between high and intermediate TCI groups, high TCI was associated with an increased risk for early NRM (d100 NRM: HR 2.05; 95% CI 1.17–3.57, *p* = 0.012; 180 NRM: HR 1.76; 95% CI 1.12–2.78, *p* = 0.015) but not for 2-year NRM (*p* = 0.19). Besides TCI category, other independent prognostic factors for NRM were incremental age, HCT-CI score ≥3, KPS score ≤80%, unrelated donor (early NRM) and a female to male transplantation (2-year NRM) (Table 3).

**Table 2:** Univariate analysis for early (d100 and d180) NRM, NRM, and REL according to TCI category.

TCI category	*N*	NRM day 100	NRM day 180	2-year NRM	2-year REL
Low	1934	4.5% [3.7–5.5]	8.2% [7–9.6]	16.5% [14.7–18.5]	29.7% [27.4–32.1]
Intermediate	1948	7.3% [6.2–8.5]	11.6% [10.1–13.1]	21.4% [19.4–23.5]	21.9% [19.8–24.0]
High	178	12.4% [8.1–17.8]	17% [11.8–23.1]	23.5% [17.2–30.5]	25% [17.9–32.6]
*P*		**0.001**	**0.001**	**0.001**	**0.001**

Results expressed as % and range. Bold denotes statistically significant.

**Table 3:** Multivariable analysis for early NRM, NRM, REL.

		NRM day 100		NRM day 180		NRM		REL	
		HR (95% CI)	*p*	HR (95% CI)	*p*	HR (95% CI)	*p*	HR (95% CI)	*p*
TCI	Low (Ref)	1		1		1		1	
	Intermediate	1.95 (1.42–2.69)	**<0.0001**	1.62 (1.26–2.08)	**0.0001**	1.44 (1.20–1.74)	**0.0001**	0.66 (0.57–0.78)	**<0.0001**
	High	4.00 (2.20–7.28)	**<0.0001**	2.86 (1.76–4.64)	**<0.0001**	1.87 (1.25–2.80)	**0.003**	0.79 (0.55–1.13)	0.20
	High vs Intermed.	2.05 (1.17–3.57)	**0.012**	1.76 (1.12–2.78)	**0.015**	1.3 (0.88–1.91)	0.19	1.19 (0.83–1.7)	0.34
Age	per 10 years	1.76 (1.31–2.37)	**0.0002**	1.66 (1.31–2.11)	**<0.0001**	1.65 (1.38–1.97)	**<0.0001**	0.95 (0.82–1.11)	0.53
HCT-CI	0 (Ref)	1		1		1		1	
	1–2	1.15 (0.81–1.62)	0.43	1.07 (0.82–1.42)	0.61	1.33 (1.09–1.62)	**0.006**	0.99 (0.83–1.18)	0.94
	≥3	1.49 (1.07–2.09)	**0.019**	1.41 (1.08–1.83)	**0.011**	1.38 (1.12–1.69)	**0.002**	1.05 (0.88–1.24)	0.59
KPS	≤80	1.65 (1.23–2.21)	**0.0008**	1.53 (1.22–1.94)	**0.0003**	1.46 (1.22–1.75)	**<0.0001**	1.02 (0.87–1.2)	0.77
Year of HCT		0.94 (0.82–1.07)	0.31	0.96 (0.87–1.07)	0.49	1.06 (0.97–1.15)	0.20	1.08 (1–1.16)	**0.045**
Sec AML	Yes vs. no	0.91 (0.64–1.29)	0.59	1.03 (0.79–1.35)	0.81	1.14 (0.94–1.39)	0.18	1.19 (1–1.41)	**0.054**
Cytogenetics	Favorable (Ref)	1		1		1		1	
	Intermediate	3.42 (0.84–13.96)	0.087	3.67 (1.17–11.54)	**0.026**	1.89 (1.00–3.56)	0.051	0.97 (0.63–1.5)	0.89
	Adverse	3.69 (0.89–15.32)	0.072	4.14 (1.3–13.2)	**0.016**	2.24 (1.17–4.29)	**0.015**	2.10 (1.35–3.26)	**0.001**
	NA/failed	4.65 (1.09–19.94)	**0.038**	5.30 (1.62–17.3)	**0.006**	2.46 (1.25–4.85)	**0.009**	1.42 (0.88–2.28)	0.15
Donor	UD vs. MSD	1.51 (1.04–2.19)	**0.031**	1.34 (1–1.79)	**0.047**	1.23 (1–1.53)	0.054	0.92 (0.77–1.09)	0.33
Sex	Female to Male	1.12 (0.78–1.61)	0.52	1.18 (0.89–1.57)	0.25	1.36 (1.11–1.66)	**0.003**	0.88 (0.72–1.07)	0.21
Graft source	PB vs. BM	1.38 (0.6–3.17)	0.44	1.08 (0.6–1.95)	0.80	0.80 (0.53–1.2)	0.28	0.64 (0.47–0.89)	0.007
In vivo TCD	Yes vs. No	1.17 (0.8–1.71)	0.41	1.20 (0.9–1.61)	0.22	1.13 (0.91–1.41)	0.26	1.14 (0.96–1.37)	0.13
Patient CMV	Pos vs. Neg	1.26 (0.91–1.75)	0.17	1.17 (0.9–1.53)	0.23	1.14 (0.94–1.39)	0.18	1.12 (0.95–1.32)	0.19
Donor CMV	Pos vs. Neg	0.77 (0.57–1.02)	0.066	0.91 (0.73–1.15)	0.44	0.95 (0.80–1.13)	0.59	1.06 (0.91–1.23)	0.43

*N* = 3791 complete cases. Cox regression models included a frailty term for center. Results are expressed as hazard ratio (HR) with 95% confidence interval (CI). Bold denotes statistically significant.

### Validation of TCI for REL

**Fig. 2:**
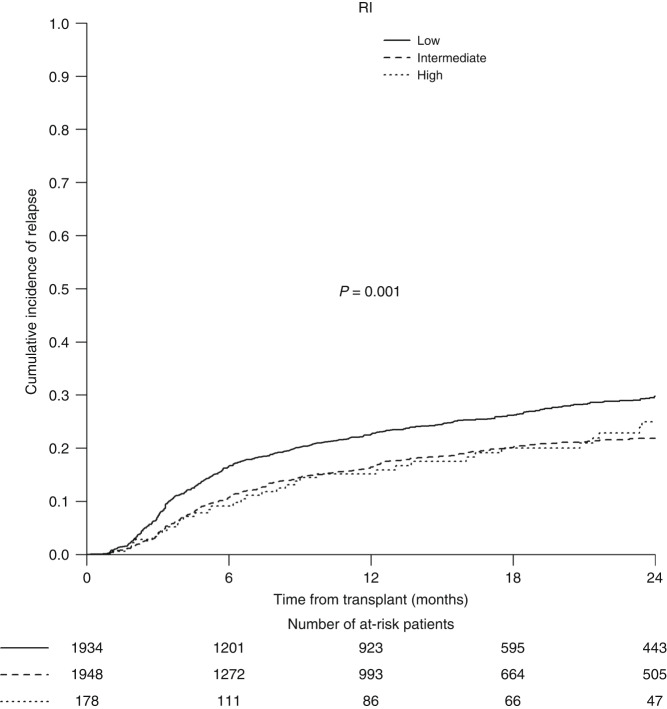
REL by TCI category. Relapse (REL) for entire validation cohort stratified by Transplant Conditioning Intensity (TCI) category (low, intermediate, high).

In univariate analysis, the REL rate was significantly higher in the low TCI group (29.7%, 95% CI 27.4–32.1) when compared to the intermediate (21.9%, 95% CI 19.8–24.0) and the high (25%, 95% CI 17.9–32.6) TCI group (*p* < 0.0001) (Fig. 2). By using the multivariable complete case analysis previously mentioned, TCI group was found to be an independent predictor for REL (Table 3). When compared with the low TCI group, the REL risk was significantly decreased in the intermediate TCI group (HR 0.66; 95% CI 0.57–0.78, *p* < 0.0001), however, we observed only a non-significant reduced REL risk trend in the recipients receiving high TCI regimens (HR 0.79; 95% CI 0.55–1.13, *p* = 0.20). REL was significantly influenced by adverse cytogenetics and the use of a bone marrow graft (Table 3). There were no significant associations between TCI group and LFS or OS (data not shown), except a borderline better OS for high versus low TCI (HR 1.35; 95% CI 1.01–1.81, *p* = 0.043).

## Discussion

To validate the original TCI, we used a cohort of more than four thousand patients transplanted in the most recent period (January 2018 to June 2021). Because allo-HCT has recently been increasingly administered to older patients and especially to those aged ≥65 years, we included in this more contemporary study patients who were one decade older (55–75 years of age) as compared to the discovery study (45–65 years) [10–12]. The chosen timeframe of the 3 most recent years is particularly useful since it includes the currently used conditioning regimens [13, 14]. In line with real-life data demonstrating a notable decrease in high dose MAC transplants over the last few years, our validation cohort included only 4% of patients being classified as high TCI, versus 21% of patients that fell into this category in the original study [15]. Taken together, this is a fully independent population and temporal validation study, reflecting present-day transplantation practice.

The TCI performed very well in this validation cohort. It stratified patients into 3 levels for early NRM, with near doubling the HR for early d100 and d180 NRM observed in each TCI group. TCI grouping provided also very strong stratification ability and independent prognostic information for 2-year NRM. The discriminative ability of TCI for NRM applies regardless of other established factors such as age, performance status (KPS), organ impairment (HCT-CI), donor type, and graft source. Of note, TCI proved to be the most important determinant of early NRM, suggesting that TCI not only stratifies conditioning intensity very efficiently but also intensity of the preparative regimen is the main driver of early NRM. Taken together, TCI could stratify the 48 different conditioning regimens used in this cohort particularly finely, based on their impact on transplant-related death, and emphasizes once again the utility of the TCI index.

Compared to TCI low regimens the use of a regimen with an intermediate TCI score was highly correlated with decreased REL, reflecting another inherently linked effect of the intensity of the preparative regimen. We found only a trend towards reduced REL risk between low and high TCI groups (HR 0.79; 95% CI 0.55–1.13, *p* = 0.20), which runs somewhat contrary to the common assumption that dose intensification may reduce relapse [16, 17]. The most plausible explanation for this finding is that the small number of recipients in the high TCI group (*n* = 178) undermined the statistical power to detect a significant effect. Moreover, opposite to the detected monotonic increase of NRM from lower to higher TCI, we found neither a significant difference nor a trend towards a reduced REL risk in the direct comparison of high versus intermediate TCI groups. Though this could again be attributed to the small sample size of the high TCI group and the low statistical power to detect differences, another explanation is that in fact the intermediate TCI group captured the so called “reduced toxicity conditioning” regimens that were specifically designed to minimize NRM without affecting REL [18]. Notably, as in the original dataset, the intermediate TCI category included in nearly equal proportion, RIC (56.4%) and MAC (43.6%) regimens. Thus, we confirm once again that although TCI was built upon the scaffolds of the MAC/RIC definitions, it represents a distinct and novel classification scheme which accounts for regimens that were not readily amenable to the RIC/MAC approach.

Transplantation is a multifactorial process, and it is a challenge to predict allogeneic HCT outcomes [18]. To account for the heterogeneity of patient and disease-specific factors, different prognostic scores for NMR (e.g. HCT-CI) or relapse risk (e.g. Disease Risk Index) have been established and constantly refined [19–22]. Likewise, the here validated TCI reflects the heterogeneity of the preparative regimens and is meant to capture in a more standardized and more precise manner their broad spectrum and to be used for risk stratification. TCI still provides valuable prognostic information for HCT outcomes but is not meant to be used for suggesting a conditioning regimen for any group of patients. Not surprisingly, the strongest prognostic information of TCI was for NRM and to a lesser extent for relapse, whereas there was no association of TCI grouping with LFS and OS. This reflects the contradictory effect of conditioning intensity in NRM and relapse and the strong likelihood of selection bias in the choice of conditioning in a retrospective study like ours. The current TCI does not account for PTCY given for GvHD prevention (used in 10.2% of patients, Table 1), which is associated with toxicities such as delayed engraftment, cardiac events, and hemorrhagic cystitis [23]. Future studies may refine and update the TCI by including to the prototype model presented here the PTCY and/or other conditioning components (e.g., antisera, novel drugs).

In summary, our study confirms in contemporary treated patients that TCI reflects the preparative regimen related morbidity, but also the anti-leukemic efficacy, highly satisfactorily and across other established prognostic factors. Though the generalizability of the model must be proven across different diseases and disease stages (except AML CR-1), ages (e.g., younger adults), and donors (e.g. mismatched), TCI index has all the features to support clinicians in their everyday clinical practice and to be instrumental in correlative analyses and comparative studies. We anticipate TCI to be used as a well-defined, easy calculated and reproducible tool to define and measure intensity of the preparative regimen before allo-HCT.

### Supplementary information

Supplementary Tables

## Data Availability

Data sharing is available through the ALWP office (myriam.labopin@upmc.fr).
