# Supplementary material for: Validation of the transplant conditioning intensity (TCI) index for allogeneic hematopoietic cell transplantation
Source: Bone Marrow Transplant. 2023 Nov 17;59(2):217–23. doi: 10.1038/s41409-023-02139-5 (PMC10849946; doi:10.1038/s41409-023-02139-5)
Supplement: Supplementary file 1 — Supplementary Tables [file 41409_2023_2139_MOESM1_ESM.docx]

**Supplementary** **Table 1. Calculation of transplant conditioning intensity (TCI) score.**

| **Component** |  | **Dose level**  **(Intensity weight)** | | | **Added points for each dose level** |
| --- | --- | --- | --- | --- | --- |
| TBI fractionated (Gray) |  | <=5 | 6-8 | >=9 | 1 |
|  | N | 323 | 57 | 13 |  |
| Busulphan (mg/kg) |  | <=6.4 iv , <=8 po | 9.6 iv , 12 po | 12.8 iv , 16 po | 1 |
|  | N | 1359 | 417 | 364 |  |
| Fludarabine (mg/m2) |  | <=160 | >=180 |  | 0.5 |
|  | N | 3726 | 210 |  |  |
| Melphalan (mg/m2) |  | 90-125 | >=140 |  | 1 |
|  | N | 263 | 474 | 1 |  |
| Cyclophosphamide (mg/kg) |  | <80 | >=90 |  | 0.5 |
|  | N | 145 | 185 |  |  |
| Carmustine (mg/m2) |  | <=250 | 280-310 | >=350 | 0.5 |
|  | N | 4 | 138 | 6 |  |
| Thiotepa (mg/kg) |  | <10 | >=10 |  | 0,5 |
|  | N | 214 | 205 |  |  |
| Cytarabine (g/m2) |  | <6 | >=6.5 |  | 0.5 |
|  | N | 45 | 177 |  |  |
| Treosulfan (g/m2) |  | 30 | 36 | 42 | 1 |
|  | N | 468 | 151 | 224 |  |
| Clofarabine (mg/m2) |  | <=150 | >150 |  | 0,5 |
|  | N | 23 | 0 |  |  |
| Etoposide (mg/kg) |  | <=50 | >50 |  | 1 |
|  | N | 3 | 0 |  |  |

Supplementary Table 1 shows intensity weighted scores for components included in transplantation conditioning regimens. The TCI score for a specific conditioning regimen was calculated by adding the intensity weights for each component given any day before the graft infusion. TCI group assignment to the low, intermediate, or high TCI category was performed according to the TCI score of [1–2], [2.5–3.5] and [4–6], respectively. For example, a regimen consisted of busulphan 12.8 mg/kg iv (3 points) and fludarabine 120 mg/m2 (0.5 points) has a TCI score of 3.5 and is assigned as an intermediate TCI regimen, whereas when the same dose busulphan is combined with cyclophosphamide 120 mg/kg as in the classical BuCy protocol the TCI score is 4 (high TCI regimen). N, number of times that this dose level of the component was used in the validation cohort. Abbreviations: iv intravenous, po *per os*, TBI total body irradiation.

**Supplementary Table 2. Conditioning regimens used.**

| **Conditioning Regimen** | **TCI low** | **TCI intermediate** | **TCI high** |
| --- | --- | --- | --- |
| *TCI score* | *[1-2]* | *[2.5-3.5]* | *[4-6]* |
| *N* | *1934* | *1948* | *178* |
| Bu1_2Cy | 6 (0.3%) | 0 (0%) | 0 (0%) |
| Bu3Cy | 0 (0%) | 5 (0.3%) | 0 (0%) |
| Bu4Cy | 0 (0%) | 0 (0%) | 57 (32%) |
| Bu2Flu | 926 (47.9%) | 0 (0%) | 0 (0%) |
| Bu3Flu | 0 (0%) | 315 (16.2%) | 0 (0%) |
| Bu4Flu | 0 (0%) | 256 (13.1%) | 45 (25.3%) |
| FluMel | 54 (2.8%) | 394 (20.2%) | 0 (0%) |
| FluMelBCNU | 2 (0.1%) | 140 (7.2%) | 2 (1.1%) |
| TreoFlu | 413 (21.4%) | 334 (17.1%) | 2 (1.1%) |
| TB2F | 152 (7.9%) | 86 (4.4%) | 0 (0%) |
| TB3F | 0 (0%) | 65 (3.3%) | 1 (0.6%) |
| TB4F | 0 (0%) | 0 (0%) | 2 (1.1%) |
| CyTBI2 | 1 (0.1%) | 0 (0%) | 0 (0%) |
| CyTBI6 | 0 (0%) | 1 (0.1%) | 0 (0%) |
| CyTBI10 | 0 (0%) | 0 (0%) | 5 (2.8%) |
| FluTBI2 | 218 (11.3%) | 0 (0%) | 0 (0%) |
| FluTBI6 | 0 (0%) | 46 (2.4%) | 0 (0%) |
| FluTBI10 | 0 (0%) | 6 (0.3%) | 0 (0%) |
| FluCyTBI | 50 (2.6%) | 2 (0.1%) | 1 (0.6%) |
| FlamsaChemo | 5 (0.3%) | 179 (9.2%) | 9 (5.1%) |
| FlamsaTBI | 0 (0%) | 25 (1.3%) | 0 (0%) |
| Bu Flu TBI2 | 0 (0%) | 5 (0.3%) | 2 (1.1%) |
| FluCy | 14 (0.7%) | 0 (0%) | 0 (0%) |
| Flu-Thiotepa | 2 (0.1%) | 0 (0%) | 0 (0%) |
| Cy-Thiotepa | 3 (0.2%) | 0 (0%) | 0 (0%) |
| BuCyFlu | 8 (0.4%) | 5 (0.3%) | 0 (0%) |
| FTT | 13 (0.7%) | 6 (0.3%) | 12 (6.7%) |
| Flu Cy Mel | 5 (0.3%) | 0 (0%) | 0 (0%) |
| Flu Mel Treo | 1 (0.1%) | 8 (0.4%) | 13 (7.3%) |
| Bu Thiotepa | 7 (0.4%) | 3 (0.2%) | 0 (0%) |
| FluMelThio | 23 (1.2%) | 37 (1.9%) | 0 (0%) |
| FBM | 0 (0%) | 14 (0.7%) | 19 (10.7%) |
| Bu Clofa | 12 (0.6%) | 0 (0%) | 0 (0%) |
| Bu CCNU | 2 (0.1%) | 0 (0%) | 0 (0%) |
| Flu-Thiotepa-BCNU | 0 (0%) | 1 (0.1%) | 0 (0%) |
| BuCy-Flu-Thiotepa | 0 (0%) | 2 (0.1%) | 0 (0%) |
| Other CT -TBI 2 | 4 (0.2%) | 6 (0.3%) | 2 (1.1%) |
| Other CT -TBI 6 | 0 (0%) | 3 (0.2%) | 6 (3.4%) |
| Other CT -TBI 9+ | 0 (0%) | 1 (0.1%) | 0 (0%) |
| Fluda alone | 4 (0.2%) | 0 (0%) | 0 (0%) |
| Fluda+BCNU | 2 (0.1%) | 0 (0%) | 0 (0%) |
| Thiotepa+Treo | 0 (0%) | 2 (0.1%) | 0 (0%) |
| CyClofa | 2 (0.1%) | 0 (0%) | 0 (0%) |
| ARAC-BuFlu | 1 (0.1%) | 0 (0%) | 0 (0%) |
| Mel-Treo | 0 (0%) | 1 (0.1%) | 0 (0%) |
| Flu-Topotecan | 1 (0.1%) | 0 (0%) | 0 (0%) |
| Mel-cladribine | 2 (0.1%) | 0 (0%) | 0 (0%) |
| Flu-Bleo | 1 (0.1%) | 0 (0%) | 0 (0%) |

**Supplementary Table 3. Cause of death.**

| **Cause of death** | **TCI low** | **TCI intermediate** | **TCI high** | **Entire cohort** |
| --- | --- | --- | --- | --- |
| *TCI score* | *[1-2]* | *[2.5-3.5]* | *[4-6]* |  |
| *N* | *629* | *631* | *69* | *1329* |
| Original disease | 312 (51.2%) | 235 (39.2%) | 26 (40%) | 573 (45%) |
| Graft vs Host Disease | 103 (16.9%) | 114 (19%) | 12 (18.5%) | 229 (18%) |
| Infection | 91 (14.9%) | 135 (22.5%) | 14 (21.5%) | 240 (18.9%) |
| Cardiac toxicity | 3 (0.5%) | 6 (1%) | 0 (0%) | 9 (0.7%) |
| Hemorrhage | 8 (1.3%) | 5 (0.8%) | 1 (1.5%) | 14 (1.1%) |
| Failure/Rejection | 1 (0.2%) | 1 (0.2%) | 0 (0%) | 2 (0.2%) |
| SOS/VOD | 1 (0.2%) | 3 (0.5%) | 2 (3.1%) | 6 (0.5%) |
| Interstitial Pneumonitis | 2 (0.3%) | 2 (0.3%) | 0 (0%) | 4 (0.3%) |
| Lymphoproliferative dis. | 1 (0.2%) | 0 (0%) | 0 (0%) | 1 (0.1%) |
| Other second malignancy | 12 (2%) | 9 (1.5%) | 0 (0%) | 21 (1.6%) |
| Multiorgan Failure | 12 (2%) | 12 (2%) | 3 (4.6%) | 27 (2.1%) |
| CNS toxicity | 4 (0.7%) | 4 (0.7%) | 0 (0%) | 8 (0.6%) |
| Other HCT-related | 26 (4.3%) | 22 (3.7%) | 4 (6.2%) | 52 (4.1%) |
| Non HCT-related | 33 (5.4%) | 51 (8.5%) | 3 (4.6%) | 87 (6.8%) |
| Missing | 20 | 32 | 4 | 56 |

**Supplementary Appendix. Contributing EBMT centers (EBMT CIC number, City [Institute])**

| **Centers (in order of patients included)** | ***Continued*** | ***Continued*** |
| --- | --- | --- |
| 235 Oslo [Rikshospitalet] | 808 Dresden [Universitaets Kl] | 124 Bochum [Knappschafts Kr] |
| 295 Hannover [Medical Univ] | 203 Leiden [Univ H] | 392 Palermo [Osp V Cervello] |
| 680 Muenster [University] | 227 Vienna [Medizinische Univ] | 589 Adana [Baskent Univ] |
| 259 Essen [Univ H] | 785 Homburg [Univ Saarland] | 693 Warsaw [Inst Haematology] |
| 552 Goettingen [Univ Kl] | 208 Zürich [208] | 746 Tartu [Univ H] |
| 786 Mainz [Johannes-Gutenberg] | 212 Stockholm [Univ H] | 749 Oldenburg [Klinikum] |
| 387 Birmingham [Queen Elizabeth] | 270 Grenoble [H A Michallon] | 134 Bonn [Uni] |
| 246 Rotterdam [Erasmus MC] | 273 Clermont-Ferrand [Jean Perrin] | 161 Tel_Aviv [Sourasky] |
| 807 Berlin [Charité Univ] | 276 Newcastle-Upon-Tyne [Freem] | 294 Milano [Osp Niguarda] |
| 524 Heidelberg [Medizinische Kl] | 338 Halle [Univ Martin-Luther] | 518 Berlin [HELIOS Kl] |
| 614 Hamburg [Univ H] | 658 Bergamo [Ospedale, ematol] | 602 Bremen [Kl Bremen-Mitte] |
| 634 Aarhus [Univ, Hematol] | 955 Amiens [H Sud] | 623 Verona [Policlinico] |
| 763 London [Kings College H] | 252 Creteil [H Mondor Hematol] | 659 Brest [C.H.R.U Brest] |
| 206 Copenhagen [Rigshospitalet] | 264 Poitiers [H La Miletrie] | 661 Rennes [H Sud/Pontchaillou] |
| 303 Cardiff [Univ Wales] | 656 Prague [Ist Hematology] | 713 Leicester [Royal Infirmary] |
| 207 Paris [St Louis] | 676 Vandoeuvre_Les_Nancy [Hosp] | 731 Umeå [Univ H] |
| 267 Pessac [H Haut-Leveque] | 705 Udine [Univ H] | 768 London [S Bartholomew`s] |
| 209 Leuven [Univ H] | 645 Marburg [Philipps Univ] | 152 Augsburg [Zentral Kl] |
| 601 Manchester [Royal Infirmary] | 809 Erlangen [University] | 214 Barcelona [H Clinic] |
| 624 Toulouse [H Purpan] | 215 Brussels [Jules Bordet] | 250 Saint_Etienne [St Etienne] |
| 671 Lyon [H E Herriot] | 283 Lund [Univ H] | 254 Leeds [St James] |
| 202 Basel [202] | 307 Rome [Univ S Cuore] | 255 Oxford [Radcliffe H] |
| 266 Uppsala [Univ H] | 598 San_Sebastian [H Aranzazu] | 397 Riyadh [King Faisal] |
| 239 Utrecht [University] | 244 Glasgow [Royal Infirmary] | 513 Munich [Kl Grosshadern] |
| 666 Villejuif [Gustave Roussy] | 251 Caen [Hopital, Hematol] | 558 Munich [Rechts der Isar] |
| 230 Marseille [Paoli Calmettes] | 289 Goeteborg [Sahlgrenska Univ H] | 594 Linz [Elisabethinen H] |
| 237 Nijmegen [St Radboud] | 302 Zagreb [Univ H Rebro] | 692 Palermo [La Maddalena] |
| 345 Haifa [Rambam MCH] | 506 Brugge [AZ Sint-Jan] | 703 Alger [P et M Curie] |
| 224 London [UCL] | 663 Valencia [H Univ La Fe] | 718 Pilsen [Charles Univ H] |
| 234 Brussels [St. Luc] | 704 Southampton [General H] | 744 Gent [Univ H] |
| 297 Frankfurt am Main [Goethe-Un] | 729 Hradec_Králové [Charles U H] | 169 Ankara [Gazi Univ] |
| 546 Groningen [Univ H] | 778 Sheffield [Royal Hallamshire] | 190 Frankfurt (Oder) [Clin Int Med] |
| 926 Montpellier [University] | 523 Nice [H de l`ARCHET I] | 284 Birmingham [Heartlands H] |
| 223 Tuebingen [Univ] | 565 Maastricht [Univ H] | 584 Barcelona [V d`Hebron Adults] |
| 261 Geneva [261] | 204 Ulm [Innere Med III] | 672 Strasbourg [H Hautepierre] |
| 277 Lille [H Claude Huriez] | 271 Innsbruck [Univ H] | 740 Linköping [Univ H] |
| 515 Helsinki [Univ Central H] | 290 Karlsruhe [Klinikum] | 769 Sevilla [Virgen del Rocio] |
| 813 Milano [S Raffaele] | 308 Graz [Medical Univ] | 780 Manchester [Christie] |
| 231 Torino [S. Giovanni (CTO)] | 561 Thessaloniki [Papanicolaou G H] | 825 Alessandria [SS Ant e Bia] |
| 386 Bristol [Royal H Sick Chil] | 588 Amsterdam [VU Univ Med Ctr] | 145 Stuttgart [Robert_Bosch_Kh] |
| 534 Cologne [Univ, Medicine] | 727 Salamanca [H Clinico] | 281 Patras [Univ H] |
| 717 Nottingham [City H] | 205 London [Hammersmith] | 309 Madrid [Jiménez Díaz] |
| 233 Besancon [H Jean Minjoz] | 225 Turku [University] | 359 Magdeburg [vGuericke U] |
| 257 Dublin [St James] | 253 Nantes [Hotel Dieu] | 390 Duesseldorf [H Heine U] |
| 644 Vilnius [Santariskiy Kl] | 299 Bolzano [Osp S Maurizio] | 544 Monza [Osp S Gerardo] |
| 810 Freiburg [University] | 348 Aachen [RWTH] | 556 Budapest [National Med Ctr] |
| 389 Leipzig [Univ, Haemat/Oncol] | 428 Gliwice [Sklodowska] | 649 Bari [Univ Studi] |
| 533 Jena [Friedrich-Schiller] | 996 Antwerp_Edegem [UZA] | 726 Liege [University] |
| 240 Bologna [S Orsola-Malpighi] | 262 Paris [Pitie-Salpetriere] | 775 Paris [St Antoine] |
| 787 Regensburg [University] | 566 Cambridge [Addenbrookes H] | 941 Rouen [Becquerel] |
| 823 Plymouth [Derriford H] | 756 Rome [Tor Vergata] | 142 Mannheim [Univ] |
| 256 Kiel [UKSH] | 811 Cagliari [Osp Businco] | 526 San_Giovanni_Rotondo [IRCCS] |

| ***Continued*** | ***Continued*** | ***Continued*** |
| --- | --- | --- |
| 640 Ljubljana [Univ Med Ctr] | 650 Angers [CHRU] | 760 Istanbul [Tip Fakueltesi] |
| 784 Essen [Evangelisches Kh] | 728 Madrid [Puerta de Hierro] | 761 Istanbul [Cerrahpasa] |
| 994 Istanbul [Nightingale] | 125 Dortmund [St. Johannes H] | 766 Napoli [Federico II] |
| 119 Ascoli_Piceno [Osp Mazzoni] | 245 Parma [Centro Trapianti] | 789 Avellino [S G Moscati] |
| 146 Stuttgart [Diakonissen Kh] | 260 Barcelona [Screu i S Pau] | 794 Perugia [Monteluce] |
| 160 Paris [H Necker] | 382 Madrid [12 de Octubre] | 868 Lecce [Osp Vita Fazzi] |
| 232 Rome [Emat, La Sapienza] | 530 Greifswald [Ernst-Moritz-Arndt] | 985 Floridablanca [FOSCAL] |
| 291 Porto [Inst Oncologia] | 559 Granada [V de las Nieves] | 993 Oran [USTO] |
| 434 Singapore [Gen H] | 580 Amman [King Hussein] |  |
| 797 Vicenza [Osp S Bartolo] | 597 Brno [Univ H] |  |
| 799 Gdansk [Medical U] | 625 Nuernberg [Klinikum] |  |
| 977 Limoges [CHRU] | 630 Brussels [Univ H] |  |
| 141 Brescia [Civili, Adulti] | 652 Tricase_(Lecce) [C Panico] |  |
| 282 Valencia [H Clinico] | 722 Palma_De_Mallorca [Son Dur] |  |
| 456 Pretoria [Albert Albert] | 864 Antalya [Medstar] |  |
| 502 Venezia [SS Giovani e Paolo] | 238 Córdoba [Reina Sofia] |  |
| 538 Wroclaw [Ctr Cell Transpl] | 304 Firenze [Careggi-Meyer] |  |
| 587 Reggio_Calabria [Centro Tr] | 339 Antwerp [AZ Stuivenberg] |  |
| 646 Roeselare [AZ Delta] | 427 Bucharest [Fundeni Clin Inst] |  |
| 665 Clamart [H Percy] | 444 Riyadh [Aziz] |  |
| 795 Pisa [Az Osp Univ] | 447 Schwerin [Helios Kl] |  |
| 919 Istanbul [Medicana Istanbul] | 627 Kayseri [Erciyes Hem] |  |
| 1006 Pavia [Fondazione IRCCS] | 889 Johannesburg [Olivedale H] |  |
| 217 Genova [S Martino] | 218 London [Royal Marsden] |  |
| 268 Belfast [City H] | 265 Milano [Osp Maggiore] |  |
| 305 Torino [Regina Margherita] | 272 Tours [H Bretonneau] |  |
| 321 Siena [Le Scotte] | 287 Rome [S C-Forlanini] |  |
| 332 Taranto [Osp Nord] | 323 Murcia [V Arrixaca] |  |
| 367 Luebeck [Schleswig-Holstein] | 421 Vigo [Comp H Univ] |  |
| 539 London [St George`s] | 616 Milano [INT] |  |
| 574 Olomouc [Univ H] | 660 Reggio Emilia [S Maria Nuova] |  |
| 613 Barcelona [H Trias i Pujol] | 677 Katowice [Silesian Med Acad] |  |
| 622 Athens [Evangelismos H] | 735 Murcia [H M Meseguer] |  |
| 648 Debrecen [University] | 759 Barcelona [H Univ Bellvitge] |  |
| 754 Tel-Hashomer [Univ Adults] | 792 Catania [Osp Ferrarotto] |  |
| 788 Ancona [Umberto I] | 819 Madrid [H G Marañón] |  |
| 970 Flensburg [St Franziskus] | 928 Salerno [Med Sch] |  |
| 143 Stuttgart [Katharinenhospital] | 104 Chemnitz [Kl Chemnitz] |  |
| 163 Piacenza [Osp Civile] | 258 Jerusalem [Univ Hadassah] |  |
| 197 Bergen [Haukeland Univ H] | 263 London [London Clinic] |  |
| 211 Sao Paulo [H Sirio-Libanes] | 364 Pune [Sahyadri] |  |
| 242 Santander [Valdecilla] | 368 Bratislava [Národný] |  |
| 248 Pescara [Osp Civile] | 529 Pesaro [O Tranpianti] |  |
| 354 Milano [Trap Mid Osseo] | 553 Krakow [Jagiellonian Univ] |  |
| 369 Beirut [American Univ] | 576 Málaga [H Carlos Haya] |  |
| 412 Ankara [Bayinder H] | 583 Riga [Haematology Ctr] |  |
| 501 Liverpool [Royal Univ H] | 585 Rostock [Kl Inn. Medicine] |  |
| 543 Modena [Policlinico] | 607 Napoli [Osp Cardarelli] |  |
| 577 Pamplona [H de Navarra] | 642 Oviedo [H Covadonga] |  |
| 606 Cuneo [S Croce e Carle] | 695 Lublin [Medical Univ] |  |
| 615 Madrid [H Ramón y Cajal] | 712 Wuerzburg [Medizinische Kl II] |  |
